# Therapeutic Potentials of Microalgae and Their Bioactive Compounds on Diabetes Mellitus

**DOI:** 10.3390/md21090462

**Published:** 2023-08-23

**Authors:** Kartthigeen Tamel Selvan, Jo Aan Goon, Suzana Makpol, Jen Kit Tan

**Affiliations:** Department of Biochemistry, Faculty of Medicine, Universiti Kebangsaan Malaysia (UKM), Jalan Ya’acob Latif, Bandar Tun Razak, Cheras, Kuala Lumpur 56000, Malaysia

**Keywords:** microalga, bioactive compounds, diabetes, hyperglycemia, metabolic disorder

## Abstract

Diabetes mellitus is a metabolic disorder characterized by hyperglycemia due to impaired insulin secretion, insulin resistance, or both. Oxidative stress and chronic low-grade inflammation play crucial roles in the pathophysiology of diabetes mellitus. There has been a growing interest in applying natural products to improve metabolic derangements without the side effects of anti-diabetic drugs. Microalgae biomass or extract and their bioactive compounds have been applied as nutraceuticals or additives in food products and health supplements. Several studies have demonstrated the therapeutic effects of microalgae and their bioactive compounds in improving insulin sensitivity attributed to their antioxidant, anti-inflammatory, and pancreatic β-cell protective properties. However, a review summarizing the progression in this topic is lacking despite the increasing number of studies reporting their anti-diabetic potential. In this review, we gathered the findings from in vitro, in vivo, and human studies to discuss the effects of microalgae and their bioactive compounds on diabetes mellitus and the mechanisms involved. Additionally, we discuss the limitations and future perspectives of developing microalgae-based compounds as a health supplement for diabetes mellitus. In conclusion, microalgae-based supplementation has the potential to improve diabetes mellitus and be applied in more clinical studies in the future.

## 1. Introduction

Diabetes mellitus is a metabolic disorder characterized by high blood sugar levels or hyperglycemia that is caused by problems with insulin secretion, insulin action, or both and it is related to carbohydrate, lipid, and protein metabolism derangements [1,2,3]. Diabetes mellitus can be categorized into type 1 diabetes mellitus (T1DM), type 2 diabetes mellitus (T2DM), and gestational diabetes [4,5]. T1DM is an autoimmune disease in which the insulin-producing pancreatic β-cells are damaged resulting in the lack of insulin production [5]. T2DM, which is the most common type of diabetes mellitus, is associated with insulin resistance and impaired insulin production. Genetic risk factors and poor lifestyle, such as physical inactivity and unhealthy diet, impose the risk of developing T2DM [4,6]. Chronic hyperglycemia induces oxidative stress that promotes the destruction of insulin-producing pancreatic β-cells, worsening hyperglycemia. Moreover, chronic low-grade inflammation contributes to the impairment in insulin signaling, thus leading to insulin resistance. Ultimately, the interplay between these factors results in a vicious cycle of hyperglycemia and progressive destruction of pancreatic β-cells. 

A study from 2019 reported that around 463 million people within the age range of 20–79 had diabetes mellitus and the prevalence was predicted to increase to 578 million and 700 million in 2030 and 2045, respectively [7]. This would negatively affect the individual’s quality of life and impose an economic burden worldwide since diabetes mellitus is one of the leading causes of premature mortality [8]. Currently, oral medication and insulin therapy are some of the strategies to manage diabetes mellitus but side effects, such as low blood glucose and gastrointestinal problems, have been reported [7,9,10]. Additionally, adopting a healthier lifestyle to improve one’s condition can be difficult; therefore, adherence to lifestyle modifications may decline over time [11,12]. Novel sources of bioactive substances that could alleviate diabetes mellitus with fewer side effects have gained traction over the years [9,13], but further exploration is needed. 

Microalgae, which include eukaryotic microalgae and prokaryotic cyanobacteria, are microscopic, unicellular, photosynthetic algae that may thrive in freshwater or saltwater [14,15]. The increased need for functional foods to improve health has led to the incorporation of microalgae into the diet [16]. In that context, microalgae biomass and their bioactive compounds have been added as ingredients in cookies, bread, mayonnaise, ice cream, soft cheeses, smoothies, olive oil, and meat products. Moreover, microalgae biomass and bioactive compound extracts are commercially available as health supplements. For example, *Chlorella vulgaris* and *Arthrospira plantensis* biomass, as well as astaxanthin from *Haematococcus pluvialis* and DHA extracted from *Schizochytrium* sp. [17]. Microalgae biomass and their bioactive compounds, such as polyunsaturated fatty acids (PUFAs), pigments, amino acids, peptides, scytonemins, pterins and phenolic compounds, have been shown to demonstrate a wide range of therapeutic effects [18]. Importantly, some of the microalgae and bioactive compounds exhibit antioxidant, anti-inflammatory, and immune-modulatory properties, and improve insulin sensitivity that could potentially alleviate diabetes mellitus [18,19,20,21]. Table 1 summarizes the bioactive compounds present in various sources that have been particularly identified in certain microalgae species and their activity on diabetes mellitus. Nevertheless, the FDA has not approved any microalgae-based products for treating or managing diabetes mellitus [22]. Therefore, clinical studies and more research are needed to confirm the anti-diabetic potential of microalgae or their bioactive compounds.

Despite the challenge, research on the anti-diabetic activity of microalgae and their bioactive compounds is increasing and their potential as health supplements for diabetes mellitus is gaining traction; however, a review summarizing the progression in this topic is lacking. Therefore, we summarized the information from in vitro, in vivo, and clinical studies investigating the anti-diabetic activity of microalgae and the bioactive compounds derived from them while explaining the mechanisms involved in producing this effect. This paper also includes the limitations and future perspectives of developing microalgae-based health supplements in managing diabetes mellitus. 

## 2. Effect of Microalgae on Diabetes Mellitus

### 2.1. In Vitro Study

Investigating the anti-diabetic effects of microalgae or their bioactive compounds in an in vitro setting is mainly focused on determining their ability to inhibit digestive enzymes that are associated with postprandial hyperglycemia or modulate enzymes or transcription factors involved in glucose metabolism (Table 2).

#### 2.1.1. Microalgae Extracts

*Arthrospira platensis*, *Nannochloropsis oculata*, *Porphyridium purpureum,* and *Chlorella vulgaris* pose potential anti-diabetic properties by inhibiting diabetic-related enzymes and alleviating oxidative stress [79]. In that context, a study reported that these microalgae extracts exhibited α-amylase inhibition [79]. One of the treatment strategies for reducing postprandial hyperglycemia is to inhibit enzymes like α-amylase and β-glucosidase to delay the absorption of glucose and control postprandial blood glucose levels [135]. In addition, it is worth noticing that every species demonstrated antioxidant potential comprising one or two free radical scavenging mechanisms; although the findings from an experiment comparing the antioxidant activities of these microalgae extracts concluded that the results vary depending on the type of assay performed. This reveals that these microalgae do not necessarily have the same underlying mechanisms in scavenging free radicals but they could alleviate oxidative stress.

The author suggested that the polyphenolic and carotenoid contents of the microalgae extracts contributed to these potential anti-diabetic properties mainly due to their antioxidant, anti-inflammatory properties, and anti-apoptotic properties [21]. Phytochemical compositional analysis revealed that all these microalgae extracts had substantial amounts of total phenolic, carotenoid, chlorophyll, and triterpenoid content. In detail, *P. purpureum* contained the highest amount of phenolic content but was limited to flavan-3-ols, whereas *A. platensis* had the most diverse phenolic content, such as flavan-3-ols, phenolic acids, and flavonols. Furthermore, *P. purpureum* and *N. oculata* had a substantial amount of carotenoids. For instance, *P. purpureum* contained carotenoids, such as zeaxanthin, β-carotene, and β-cryptoxanthin. As for the total chlorophyll content, *A. platensis* had the highest chlorophyll content followed by *N. oculata*, *C. vulgaris,* and *P. purpureum*. As for the triterpenoid content, *A. platensis* had the highest total content which was markedly higher compared to the other species.

Additionally, another in vitro study to measure the inflammation biomarker myeloperoxidase (MPO) release from human neutrophils revealed that *A. platensis* decreased the MPO release from human neutrophils in a dose-dependent manner [136]. MPO is involved in inflammation and oxidative stress, and its level is correlated to diet-induced insulin resistance and obesity [137]. Therefore, the results from this study further confirm that *A. platensis* exerts anti-inflammatory properties in alleviating hyperglycemia from an in vitro standpoint. A study comparing glucosidase inhibition among microalgae species isolated from the Amazon revealed that *Synechococcus* sp. had one of the highest glucosidase inhibitions, thus emerging as a potential anti-diabetic supplement [134]. In that context, the highest concentration of *Synechococcus* sp. methanolic extract exhibited 90.4% and 96.9% inhibitory activity against α- and β-glucosidase, respectively. The author suggested that the glucosidase inhibition by the microalgae extracts could be due to the pigments in them, such as purified C-phycoerythrin (C-PE) and phycocyanin [138]. Moreover, their extracellular and intracellular polysaccharides could play a part in achieving anti-diabetic effects [139].

#### 2.1.2. Protein Hydrolysate

Villaro et al. investigated the antioxidant and anti-diabetic properties of enzymatic hydrolysates derived from the phycobiliproteins of *A. platensis* [126]. Proteins isolated from *A. platensis* could be hydrolyzed into potential bioactive peptides exhibiting antioxidant, dipeptidyl peptidase-IV (DPP-IV), and α-amylase and α-glucosidase inhibitory properties. Inhibition of DPP-IV improves blood glucose fluctuations and enhances glycemic control in diabetes. Blood glucose fluctuations are linked to pancreatic β-cell impairment; therefore, DPP-IV inhibition is an effective strategy to alleviate diabetes [140,141,142]. DPP-IV inhibition controls postprandial insulin secretion by increasing the levels and extending the effects of incretin hormones, namely, glucagon-like peptide 1 (GLP-1) and gastrointestinal insulinotropic peptide (GIP) that regulate insulin secretion in response to nutrients [141,143]. Additionally, it promotes the proliferation of β-cell by inhibiting apoptosis [140]. Results showed that pepsin and ficin hydrolysates had the highest antioxidant capacities compared to other hydrolysates. As for DPP-IV inhibition, alcalase hydrolysates showed the highest capacity compared to other hydrolysates. However, all the tested hydrolysates happened to have limited α-amylase and α-glucosidase inhibitory capacities. The authors suggested that perhaps the concentration used in this experiment was quite low to produce any significant α-amylase and α-glucosidase inhibition.

#### 2.1.3. Oxo-Fatty Acids

Oxo-fatty acids (oFAs), a type of long-chain fatty acid, have been shown to demonstrate peroxisome proliferator-activated receptors (PPARs) agonist activities to regulate energy metabolism [144]. PPARs, such as PPARγ and PPARα, are a family of ligand-dependent transcription factors which function to modulate the expression of genes involved in lipid and glucose metabolism [145]. In that context, PPARγ and PPAR-β/δ activation improves insulin sensitivity and modulates lipid metabolism, respectively [146]. Therefore, targeting PPARs is a common and effective approach to managing diabetes mellitus [132]. Sæther et al. investigated the PPAR agonist activity of oFAs, namely, [(7E)-9-OHE] and [(10E)-9-OHE] derived from *C. karianus* in hepatocytes and adipocytes [132]. These oFAs exhibited substantial PPAR agonist activity in a dose-dependent manner. Next, the endogenous PPAR target genes activation was studied by treating Huh-7 cells and SGBS adipocytes with oFAs. Generally, it was found that the treatment activates fatty acid catabolism by upregulating CPT1A, ACSL3, PLIN1, and ANGPTL4 gene expressions. CPT1A, ACSL3, and PLIN1 genes function to regulate lipid metabolism [147,148], whereas ANGPTL4 gene regulates energy metabolism, and its upregulation has been reported to improve hyperglycemia in diabetic mice [149,150]. Overall, this result suggests that oFAs modulate energy metabolism in the hepatocytes and adipocytes.

Moreover, the adipogenic potentials of oFAs were studied via adipocyte transcriptomic analyses. Although the focus was to compare the dyslipidemic activities of oFAs, the authors reported that oFAs enhanced insulin-sensitizing adiponectin and leptin gene expressions while suppressing pro-inflammatory cytokines gene expressions due to PPARγ activation. Moreover, IRS1 and SLC2A4 genes were upregulated resulting in improved adipose insulin sensitivity [151,152]. Therefore, the activation of anti-diabetic gene programs is achieved by decreasing pro-inflammatory cytokines and raising insulin-sensitive adipokines. As a result, [(7E)-9-OHE] and [(10E)-9-OHE] derived from *C. karianus* are referred to as semi-potent dual PPARα/γ agonists with the potential to alleviate hyperglycemia.

### 2.2. In Vivo Studies

Several studies have evaluated the effects of microalgae or their bioactive compounds on a well-established diabetic rodent model characterized by high blood glucose levels. Table 3 summarizes the findings and the mechanisms involved in alleviating diabetes mellitus. 

#### 2.2.1. *Arthrospira platensis*

Studies have indicated that *A. platensis* or biomass supplementation alleviates hyperglycemia in diabetic rats mainly due to its antioxidant and anti-inflammatory activities [161,162]. Nasirian et al. supplemented streptozotocin (STZ)-induced diabetic male Wistar rats with 10, 20, or 30 mg/kg body weight (BW) per day of *A. platensis* extract for 3 weeks [157]. A total of 20 and 30 mg/kg of extracts decreased glucose, triglyceride (TG), cholesterol (TC), and low-density lipoprotein cholesterol (LDL-C) levels that were elevated in diabetic rats. Improvement in hyperglycemia and hyperlipidemia was attributed to the trace minerals (TMs) present in *A. platensis* extract. Chemical compositional analysis confirms that the extract contains zinc (Zn), copper (Cu), iron (Fe), selenium (Se), chromium (Cr), and magnesium (Mg). According to studies, TMs provide insulin-modulatory effects [163]. In addition, TMs potentiate antioxidant activity in diabetes [164]. These doses also increased superoxide dismutase (SOD), glutathione peroxidase (GSH-Px), and catalase (CAT) enzyme concentrations, thus explaining the lowered serum malondialdehyde (MDA) concentrations. The author explains that Zn and Se regulate antioxidant enzyme activities by being a cofactor for SOD and participating in antioxidant reactions, respectively [165,166]. In addition, Cr and Mg play a role in regulating glucose metabolism due to their involvement in insulin signaling [167,168,169]. Moreover, Mg deficiency has been linked to insulin resistance [167,168]. Furthermore, the highest dose decreased plasma concentrations of TNF-α and IL-6. These results imply that the anti-inflammatory effect is related to the antioxidant activities of *A. platensis* extract. Therefore, TMs improve glucose metabolism and reduce oxidative stress and inflammation.

The anti-inflammatory property of *A. platensis* has been shown to be involved in the protection of pancreatic β-cells. Hyperglycemia causes reactive oxygen species (ROS)-mediated glucose toxicity in pancreatic β-cells that impairs their insulin-producing function, leading to insulin insufficiency [170]. Alloxan-induced diabetic male Wistar or Swiss rats were supplemented with 25, 50, or 100 mg/kg/day (d) of *A. platensis* powder for 5 or 10 days [136]. It was reported that the 100 mg/kg dose restored the pancreatic islet area and β-cell characteristic and decreased pro-inflammatory TNF-α levels. A separate experiment revealed that 5, 10, and 25 mg/kg doses reduced mice licking time in a formalin test substantially, thus being denoted as anti-inflammatory. Moreover, 50 and 100 mg/kg doses decreased edema, improved tissue architecture, and reduced neutrophil infiltration in the paw tissues of mice. Importantly, serum glucose levels decreased especially on the 10th day. Furthermore, these doses decreased serum TG and TC in a dose-dependent manner. Therefore, these results agree on the anti-inflammatory effects of *A. platensis* and its role in alleviating hyperglycemia by regulating pro-inflammatory cytokine levels.

In addition, *A. platensis* extract improves glucose tolerance in diabetic rats by positively affecting the gut microbiota composition. Moreover, different extract types seem to exert different anti-diabetic effects. In that context, male rats that were induced diabetes by a high-fat high-sucrose diet were supplemented with 150 mg/kg/day of ethanol or water extracts of *A. platensis* for 8 weeks [43]. The results show that the fasting blood glucose (FBG) level was significantly lower in diabetic rats supplemented with water extract of *A. platensis* compared to non-supplemented diabetic rats. Moreover, an oral glucose tolerance test (OGTT) revealed that both extracts improved glucose tolerance. Interestingly, gut microbiota composition, which was also improved mainly by increasing the abundance of *Oscillibacter,* was indicated to play a crucial role in attenuating the adverse conditions seen in diabetic rats. These results suggest that *A. platensis* supplementation, especially its water extract, is efficacious in alleviating hyperglycemia, the hallmark of diabetes mellitus. The author explains that this could be attributed to PUFAs in their extracts that may modulate energy metabolism and improve pancreatic β-cell functions [171]. In addition, it may improve the gut microbiota composition to prevent low-grade chronic inflammation and insulin resistance [172]. In this study, the slight yet significantly higher efficacy of water extract over ethanol extract indicates that some of the bioactive compounds could better dissolve in water compared to ethanol.

Moreover, *A. platensis* supplementation could modulate energy metabolism by regulating the enzymes involved in glucose metabolism. Furthermore, this supplementation exerts anti-apoptotic properties to protect pancreatic β-cells. Supplementing 500 mg/kg of *A. platensis* powder twice a week for 2 months to STZ-induced diabetic male Albino rats decreased serum glucose and glycated hemoglobin (HbA1c) concentrations and increased serum insulin concentration [159]. This study revealed that supplementation decreased pyruvate carboxylase (PC) mRNA expression. PC is a crucial gluconeogenic enzyme and its increase due to insulin deficiency may be linked to hyperglycemia [173,174]. The author suggests that the glucose-lowering effect of *A. platensis* may be due to its ability to inhibit PC expression. Furthermore, supplementation restored the architecture of pancreatic islets and increased glutathione concentration. Moreover, the activities of glutathione-S-transferase (GST), SOD, and catalase were increased. The upregulation of SOD, catalase, and GST mRNA expressions in these rats further corroborates those findings. These enzymes counteract the effects of ROS by neutralizing them to protect pancreatic β-cells [175]. Therefore, this explains the decrease in MDA and TBARS concentrations which are lipid peroxidation biomarkers. The antioxidant activity is credited to the phytochemicals such as phenolic compounds, namely, caffeic acid, coumaric acid, gallic acid, catechin, apigenin, flavonoids, flavonols, and phycocyanin that have antioxidant potential [159]. Moreover, *A. platensis* supplementation decreased pro-apoptotic Bax, CASP-3, and pro-inflammatory TNF-α mRNA expressions and increased anti-apoptotic Bcl-2 mRNA expression. Additionally, p-p38, p-ERK, and p-JNK MAPKs protein expressions decreased, showing that the activation of p38- and ERK-mediated MAPK signaling was inhibited. A crucial element of the pro-apoptotic signaling pathway imposed on diabetes is the MAPK signaling cascade [176]. MAPK activation may be mediated by ROS and its repression is credited to the antioxidant properties of *A. platensis*.

#### 2.2.2. *Nannochloropsis oculata*

*N. oculata* supplementation was reported to have corrected hyperglycemia and hyperlipidemia in diabetic rats. A total of 10 or 20 mg/kg/day of *N. oculata* supplementation for 3 weeks on STZ-induced male Wistar diabetic rats resulted in increased BW, serum insulin, and high-density lipoprotein cholesterol (HDL-C), and decreased serum glucose, TG, TC, and LDL-C concentrations [156]. However, the underlying mechanism was unexplored in this study. The authors suggest that the antihyperglycemic properties of *N. oculata* could be due to its protective effects against oxidative damage on pancreatic β-cell islets or enhanced glucose uptake by peripheral tissues which is comparable to insulin therapy on STZ-induced diabetic rats [21,177]. The pigments in *N. oculata* could have exerted the previously mentioned antioxidant effects to counteract the oxidative damage caused by STZ on pancreatic islets [178]. Moreover, Se in *N. oculata* was suggested to play a role in regulating the antioxidant defense system [163,179]. The improvement in lipid profile could mean that lipid metabolism was improved as well as glucose metabolism, mainly by regulating insulin secretion and action. Additionally, the EPA and DHA in *N. oculata* could have played a role in improving the lipid profile [180].

Another study with a similar study design and doses of *N. oculata* powder has proven its antihyperglycemic effect by regulating antioxidant enzymes and pro-inflammatory cytokine levels [153]. First, the supplementation increased BW in a dose-dependent manner. The underlying mechanism of this effect is largely unknown, but the authors speculate that it could be due to an improvement in insulin sensitivity and glycemic control [181,182,183]. This was corroborated when serum glucose and insulin in supplemented diabetic rats were also improved in a dose-dependent manner. In addition, serum concentrations of GSH-Px, SOD, and FRAP were increased and serum concentration of MDA was decreased in supplemented rats which suggests the anti-antioxidant activity of *N. oculata*. Furthermore, serum concentrations of pro-inflammatory markers, such as IL-6, NF-κB, IL-1β and TNF-α, were significantly decreased. Thus, the anti-inflammatory activity of *N. oculata* could also be credited for the improvement in hyperglycemia in diabetic rats. The authors suggest that carotenoids present in *N. oculata* could have exerted these bioactivities [178].

#### 2.2.3. *Nannochloropsis gaditana*

Similar to *N. oculata*, 10% of *N. gadiatana* powder supplementation per day for 2 months on STZ-induced diabetic male Wistar rats also improved BW and serum glucose [2]. Furthermore, this study reported an improvement in HbA1c. Additionally, *N. gaditana* attenuated glycosuria and polyphagia which marked its potential in reversing diabetic complications [184,185]. These effects were credited to the modulatory effect on pancreatic β-cell insulin secretion, thus improving glucose metabolism. In addition, the serum TC and TG levels were decreased in diabetic rats upon *N. gaditana* supplementation, which could be due to improvement in overall energy metabolism exerted by modulating insulin secretion. Furthermore, it was reported that CAT, glutathione, and SOD enzymes were increased in the liver mitochondria and pancreatic tissues. Moreover, CAT, glutathione, SOD, and GST in the liver tissues were increased. Therefore, the liver mitochondrial MDA and carbonyl proteins concentrations were also decreased. Carbonyl proteins are markers of protein oxidation similar to how MDA is for lipid peroxidation, and these reflect the oxidative status in the rats [186]. It was mentioned that the overwhelming levels of ROS in the mitochondria lead to oxidative stress, and this could underlie the mechanism of diabetes induction by STZ [187,188,189]. Previous studies have reported that *N. gaditana* scavenges free radicals and prevents lipid peroxidation [190]. Furthermore, the serum concentrations of IL-6 and TNF-α were reduced. This effect was attributed to the bioactivity of carotenoids in *N. gaditana* [191,192].

#### 2.2.4. *Chlorella pyrenoidosa*

According to a study, *C. pyrenoidosa* exerts its anti-diabetic effects by modulating energy metabolism and improving gut microbiota composition [43]. In that context, water and ethanol extracts of *C. pyrenoidosa* were supplemented to the diet-induced diabetic male rats. Both extract types decreased FBG and improved glucose tolerance denoted by the decreased area under the curve (AUC) in the OGTT. In terms of its effects on the composition of gut microbiota, *C. pyrenoidosa* restores the microflora balance of the intestine. The supplementation decreased the Firmicutes/Bacteroidetes ratio which is said to be a sign of improvement in the microbiota of the intestine and chronic low-grade inflammation [193,194]. Moreover, some bacterial species that were imbalanced in the gut microbiota of non-supplemented diabetic groups were reversed in the supplemented groups. For example, supplementation decreased *Blautia* and *Turicibacter* compositions. *Turicibacter* is said to cause diabetes and inflammation [195]. Moreover, beneficial bacteria, such as *Oscillibacter*, *Parasutterella*, and *Ruminococcus* species, were maintained by the supplementation. *Ruminococcus* and *Oscillibacter* seemed to negatively correlate with AUC FBG. However, these findings are not consistent with other studies and there is a lack of extensive characterization of the gut microbiota in diabetic rat models [196,197]. At every parameter, *C. pyrenoidosa* performed better than *A. platensis* suggesting that the former is more efficacious at exerting anti-diabetic effect. The author speculates that the improvement in the abundance of *Ruminococcus* could underlie the increased hypoglycemia effect of *C. pyrenoidosa* compared to *A. platensis*. These effects could be attributed to polyunsaturated fatty acids (PUFAs) found in *C. pyrenoidosa* [198,199].

#### 2.2.5. *Porphyridium cruentum*

A study has demonstrated the therapeutic effect of *P. cruentum* powder on diabetes due to its protective and proliferative effects on pancreatic β-cell islets. STZ-induced male Sprague–Dawley rats that were supplemented with 600, 1200, and 1800 mg/kg/day for 14 days had increased β-cell granulation and the number of pancreatic β-cells. Additionally, 1200 and 1800 mg/kg doses increased pancreatic islets of Langerhans area, but these parameters were markedly reduced by diabetes. The pancreatic β-cells produce and synthesize insulin that most importantly regulates blood glucose levels, and its impairment leads to a lack of insulin synthesis and subsequent metabolic control, leading to hyperglycemia [200]. An increase in the number of pancreatic β-cells may result in enhanced insulin secretion and cell regeneration, which lower blood sugar levels. Therefore, therapeutics targeting the enhancement of pancreatic β-cell regeneration could alleviate diabetes [201,202]. Previous studies have reported the presence of bioactive compounds, such as phycobiliproteins, carotenoids, extracellular polysaccharides, B-phycoerythrin (PE), and omega-3 fatty acids, that could exert antioxidant and anti-inflammatory properties which may attenuate hyperglycemia [67,82,203,204]. Nevertheless, *P. cruentum* supplementation did not alleviate blood glucose levels of the diabetic rats suggesting that the doses used in this study may not be optimum to impart any hypoglycemic effect.

#### 2.2.6. *Dunaliella salina*

Ruperez et al. investigated the effects of *D. salina* extract on the metabolism of STZ-induced male Sprague–Dawley rats [160]. The diabetic rats received 150 mg/kg of *D. salina* extract at 72, 64, 48, 40, and 24 h before sacrifice. Metabolic fingerprinting, a comprehensive analysis of metabolic profiles, was performed to assess any changes caused by the extract. The results of the study revealed significant alterations in the metabolic profiles of the diabetic rats after receiving *D. salina* extract. This is due to the fact that the urine fingerprinting PLS-DA scores plot shows a clear separation between supplemented and non-supplemented groups. The extract demonstrated a positive effect on most of the parameters measured, indicating potential therapeutic properties for diabetes management. In that context, there was a slight increase in BW and a decrease in liver size. The author speculates that the extract could have provided better metabolic control resulting in increased liver glycogen utilization which is a sign of improvement in Mauriac syndrome in type 1 diabetes [205]. In addition, supplementation decreased TBARS, increased antioxidant enzymes such as reduced glutathione (GSH), and oxidized glutathione (GSSG), and decreased GSSG/GSH ratio in the liver but not in the blood. The glutathione antioxidant system is dysregulated or imbalanced due to increased ROS production induced by hyperglycemia that leads to insulin resistance and pancreatic β-cell death [206,207,208]. These results show that *D. salina* has antioxidant properties by reducing markers of oxidative degradation and increasing antioxidant capacity exclusively in the liver. Moreover, a-tocopherol in plasma was increased suggesting that *D. salina* could protect against damage from free radicals [209]. Furthermore, *D. salina* has been reported to contain β-carotene and bioactive peptides that exhibit antioxidant activities. Nevertheless, the supplementation did not improve blood glucose levels in this study, but the TG levels were improved. This suggests that the extract exerts an antihyperlipidemic function that could alleviate complications associated with diabetes mellitus. Overall, the findings suggest that *D. salina* extract has the potential to positively influence the metabolism of diabetic rats.

#### 2.2.7. Eicosapentaenoic Acid (EPA) and Docosahexaenoic Acid (DHA)

Administration of omega-3 fatty acids has been reported to improve diabetes by regulating lipid metabolism, insulin synthesis, and attenuating oxidative stress and insulin resistance by modulating the expressions of inflammatory and energy metabolism genes [210,211,212,213]. Microalgae are well-known as a good source of omega-3 fatty acids, namely, eicosapentaenoic acid (EPA) and docosahexaenoic acid (DHA) [214]. Genetically diabetic male db/db mice supplemented with 1 mg/g of EPA and DHA extracted from *Chlorophyceae* and *Eustigamatophyceae* families enhanced their total antioxidant capacity in adipose tissue and plasma [155]. However, the supplementation could not manage to attenuate the increase in lipid peroxidation observed in diabetic mice. Nevertheless, the author explains that previous studies have reported successful alleviation in MDA and promotion of the synthesis of antioxidants in the liver and platelets upon supplementation of microalgae fatty acids [215]. Meanwhile, diabetic rats fed a rodent chow enriched with 2.0% microalgae EPA and DHA had decreased food intake. This effect could be due to the regulatory effect of the fatty acid supplementation on adiponectin synthesis. Adiponectin is an adipokine released by adipose tissue that has been demonstrated to improve insulin sensitivity, control inflammation, and regulate appetite [216,217].

Another study found that supplementation with microalgae EPA and DHA extracted from *Chlorophyceae* and *Eustigmatophyceae* families on db/db mice at similar dosages had profound anti-inflammatory properties [158]. In that context, T lymphocyte populations decreased. T lymphocytes have been reported to play a role in the inflammatory processes and adipose tissue insulin resistance underlying the pathogenesis of diabetes mellitus [218,219]. Furthermore, diabetic rats supplemented with microalgae-derived n-3 fatty acids caused a decrease in the levels of pro-inflammatory cytokines IFN-y and TNF-α. Nevertheless, the IL-12 pro-inflammatory cytokines were increased by the fatty acids supplementation. Additionally, supplemented diabetic mice had an increase in anti-inflammatory IL-10 and TGF-β cytokine levels. Moreover, supplemented groups had the least increase in IL-6 cytokine levels compared to other groups. Therefore, microalgae-derived EPA and DHA supplementation exerts a modulatory effect on cytokine levels and the T lymphocyte population, thus improving inflammation in diabetes mellitus.

#### 2.2.8. Polysaccharides

Microalgae-derived polysaccharides have been studied for their antihyperglycemic activity since it slows down gastric emptying and the absorption of glucose in small intestines and protects pancreatic β-cells [82,220,221]. High-glucose high-fat diet, D-gal, and STZ-induced male Kunming diabetic mice supplemented with 150 or 300 mg/kg of *C. pyrenoidosa* polysaccharides (CPP) for 4 weeks were observed to have improved BW and insulin levels [154]. Additionally, glucose uptake was enhanced in mice supplemented with the higher dosage, performing better than metformin which was included as a positive control in this study. This shows that CPP improves glucose metabolism attributed to their antioxidant activity suggested by the increase in antioxidant enzymes, such as SOD, CAT, and GSH-Px enzymes in tissues and reduction in MDA levels upon supplementation. CPP supplementation also imparts protective effects on tissues that were observed to be injured in AD mice due to STZ and D-gal. In that context, CPP improved pancreatic architecture that could improve β-cell insulin secretion and sensitivity [222]. Additionally, CPP supplementation demonstrated anti-inflammatory effects by decreasing IL-6R mRNA and protein expressions. Moreover, the lower dosage decreased forkhead box O1 (FOXO-1) and increased glucagon-like peptide-1 receptor (GLP-1R). FOXO-1 is a nuclear transcription factor that is involved in mediating insulin levels and current literature suggests that it could be a therapeutic target in alleviating T2DM [223]. Moreover, GLP-1R plays a role in regulating blood glucose levels by enhancing insulin secretion; therefore, its upregulation could alleviate hyperglycemia [224]. Ultimately, the co-modulation of IL-6R/FOXO-1 and GLP-1R/FOXO-1 pathways are suggested to be the underlying mechanisms for the effects imparted by CPP supplementation. A metabolic profiling analysis revealed that phenylpyruvic acid was reduced, correlating with the improvement in glucose uptake.

In another study, the administration of extracellular polysaccharides (Eps) derived from *P. cruentum* at 150, 300, and 450 mg/kg/day for 14 days on STZ-induced male Sprague–Dawley rats decreased food intake [82]. Furthermore, 300 and 450 mg/kg of EPs managed to reduce blood glucose levels, being as effective as glibenclamide. Apart from slowing down intestinal glucose absorption, EPs may also regulate the secretion of insulin to control blood glucose levels. In addition, supplementation, especially at the highest dosage, improved pancreatic islets’ area and the number of pancreatic β-cells, proving its protective effect against STZ-induced damage on the pancreas. STZ enters the β-cells via glucose transporter 2 (GLUT2) receptors which then induces its toxicity on the pancreas, but EPs seem to compete with STZ for GLUT2 receptors which could explain the protective effect and the attenuation of blood glucose levels [225,226,227]. The author also speculates that this could be due to reduced cytotoxic nitric oxide (NO) and regulating nitric oxide synthase (iNOs) enzyme gene expressions. The highest dosage also improved intestinal villi height similar to fiber supplementation that stimulated the secretion of GLP-2 hormone which is responsible for increasing nutrient absorption and developing and maintaining an optimum gastrointestinal tract condition [228,229]. The results suggest that EPs derived from *P. cruentum* could be a viable option in managing diabetes.

#### 2.2.9. Astaxanthin

Microalgae pigments, such as chlorophylls, carotenoids (β-carotene and astaxanthin), and phycobilins (phycocyanin, phycoerythrin, and phycoerythrocyanin) are photosynthetic pigments commonly used as dyes in the food, cosmetic, and pharmaceutical industries [230,231]. By protecting pancreatic β-cells, improving insulin resistance (IR), and enhancing insulin secretion, astaxanthin is said to lower blood glucose levels [54,232,233]. Genetically diabetic db/db female mice supplemented with 1.0 mg/mouse/day of astaxanthin derived from marine microalgae for 12 weeks decreased non-fasting blood glucose, improved intraperitoneal glucose tolerance (IPGT), and increased serum insulin levels [55]. Therefore, these results suggest that astaxanthin could preserve pancreatic β-cell function. The author explains that this protective effect is due to the improvement in oxidative stress caused by ROS in the pancreas which is prone to oxidative damage caused by hyperglycemia. Additionally, hyperglycemia leads to the production of advanced glycosylation end products (AGEs), which together with ROS leads to impairment in insulin gene transcription and pancreatic β-cell death [234].

A study carried out by Penislusshiyan et al., investigating the in vitro antioxidant activity of astaxanthin derived from *H. pluvialis,* explains its mechanism of action [235]. In that context, astaxanthin exhibited significant antioxidant activity attributed to its polyene chain and the C3 methine at its terminal ring moiety [231,236]. Moreover, in silico interaction analysis of astaxanthin with S. cerevisiae α-glucosidase revealed that it interacts with the catalytic site amino acid residue of S. cerevisiae α-glucosidase by hydrogen bonding that indicates their interaction with the active site of this enzyme [235]. The result also reveals that the polyene chain of astaxanthin interacts with a few amino acids at the active site of S. cerevisiae α-glucosidase by hydrophobic interaction. Moreover, astaxanthin interacts with active sites of human α-glucosidases. However, astaxanthin could be viewed as a precursor molecule that could be conjugated with other candidate compounds to increase its efficacy and potency in terms of enhancing its antioxidant activity and the number of interactions to α-glucosidases [235].

### 2.3. Human Studies

There have not been many well-designed clinical trials examining the effects of various microalgae-based supplementations on diabetic human subjects. In addition, the trials are limited to T2DM patients which could be due to the potential of microalgae or its bioactive compounds in alleviating metabolic disorders. Table 4 summarizes the findings from the human studies.

#### Microalgae Extract

*A. platensis* supplementation has been shown to improve the glycemic profile of patients with T2DM. Doses of *A. platensis* as low as 1 or 2 g and/or as high as 8 or 14 g per day improved FBG and glycated protein levels such as HbA1c [237,238,239,240,241]. Moreover, *A. platensis* supplementation improved lipid profile which is often deranged in diabetics leading to cardiovascular diseases and death [237,238,239,242]. However, the anti-diabetic effects are not dose-dependent which could be due to variations in the severity of diabetes in the patients. In addition, the durations of supplementation were not standardized among the studies and were rather short in certain studies. This limitation could explain the insignificant changes in HbA1c levels in some of these studies since HbA1c is known to take a longer duration for changes to appear. Therefore, the results, although seem promising, are not comparable to one another. Nevertheless, even the highest dosage and the longest test duration did not induce toxicity or side effects on the patients, suggesting its safety to be included as a health supplement.

Furthermore, a study has reported that *A. platensis* demonstrates a synergistic anti-diabetic effect with metformin while improving lipid profiles in T2DM patients [242]. A total of 2 g of *A. platensis* administered together with their daily metformin regimen for 3 months managed to decrease the HbA1c, FBS, TC, LDL-C, and TG, and increase the HDL-C levels better than metformin alone. This suggests that *A. platensis* supplementation could be an additional strategy for managing diabetes without the side effects associated with anti-diabetic drugs.

The antihyperglycemic properties of *A. platensis* could be attributed to the upregulation of a crucial insulin regulatory mechanism, namely, the adenylate cyclase/cAMP pathway which causes increased cAMP levels followed by upregulation of PKA activity leading to insulin secretion [242]. Moreover, pigments, such as pheophytin, β-carotene, and phycocyanobilin, could inhibit important enzymes associated with diabetes, such α-amylase, α-glucosidase, G6PD, and DPP-IV, thus regulating their activity and attenuating hyperglycemia [7].

## 3. Limitations

Applying microalgae and their bioactive compounds as health supplements to alleviate diabetes mellitus poses certain limitations. First, only one research has been carried out for most microalgae species or their bioactive compounds. Additionally, the effects of other potential microalgae species or their bioactive compounds on diabetes mellitus are unexplored. Moreover, some of the studies employed only a single dosage of microalgae or their bioactive compounds to assess their anti-diabetic properties. Furthermore, some microalgae dosages were selected based on previous studies on different microalgae species which may not have similar bioactive compound composition or concentration. Therefore, the optimum dosage for most of the test compounds is largely unknown and there is no clear verdict as to how efficacious these dosages are if they were to be considered as health supplements. In addition, the method of inducing diabetes mellitus in animal models varies among studies; therefore, the therapeutic effects of the test compounds cannot be generalized for every type of diabetes. Moreover, the disease animal models may not completely recapitulate the complex pathophysiology of diabetes, especially T2DM. Furthermore, the anti-diabetic mechanisms were only elucidated in some of the studies. Additionally, information on the digestibility or bioavailability of the specific microalgae extracts is lacking, which is crucial since an intact microalgae cell wall could hamper the bioavailability and lessen the potency of the bioactive compounds. Finally, some of the clinical trial study designs face limitations in terms of small sample size, no placebo groups, and short duration of intervention.

Moreover, comprehensive data on the safety of microalgae consumption are not available yet. Extensive toxicological studies are limited to only some species and their bioactive compounds, especially the ones that are commonly consumed, such as *A. platensis* and astaxanthin, which have been reported as safe for consumption by governmental bodies [243,244]. Generally, *A. platensis* does not contain toxins, deeming it safe for consumption [45,243,245]. Many in vivo studies include liver enzyme analysis to elucidate the basic toxicity of microalgae or its bioactive compounds. For example, *N. oculata*, *N. gaditana*, *D. salina* and *C. vulgaris* extracts, fucosterol, and EPA from *N. oculata* have been reported to be safe in preclinical studies [246,247,248,249,250]. However, there is a lack of evidence on the safety of higher dosages or long-term consumption on human subjects; therefore, their optimal dosage is still inconclusive. Additionally, contamination due to the accumulation of heavy metals from the environment or toxins produced by cyanobacteria in the culture could result in toxicity [45,251]. As a result, adverse effects upon consumption of certain microalgae have been reported [45]. Therefore, the safety of a particular microalgae depends on its source, culture conditions, and quality regulation.

## 4. Future Perspectives

**Figure 1:**
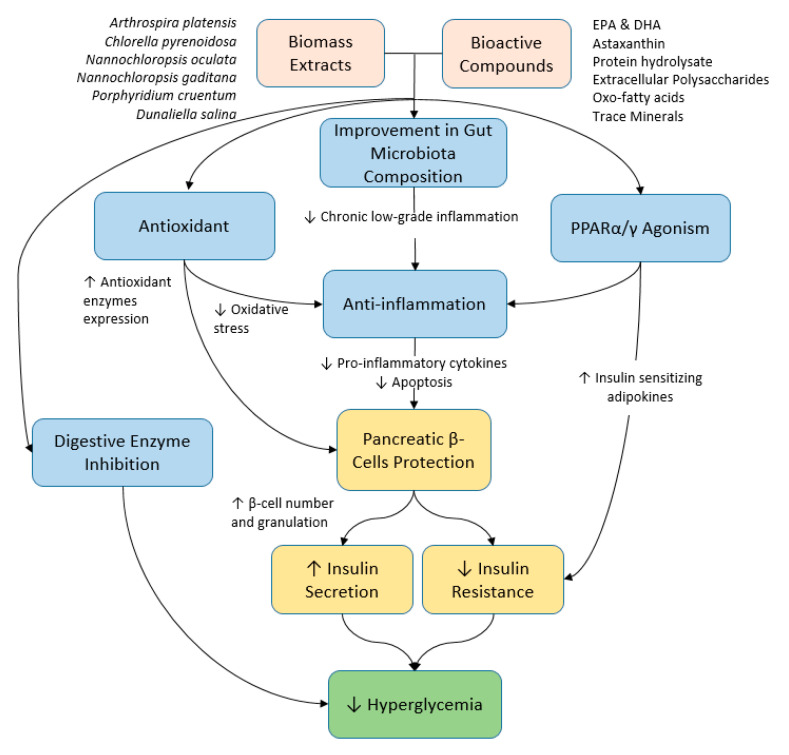
Summary of the potential therapeutic effects and the mechanisms of microalgae and their bioactive compounds on hyperglycemia.

More studies are needed to elucidate the underlying anti-diabetic mechanisms of microalgae and their bioactive compounds by means of studying the genes and proteins involved. In addition, future studies shall employ omics technology to gain valuable insights into the molecular changes imparted by a particular microalga or its bioactive compounds. In that context, novel biomarkers, therapeutic targets, and upregulated or downregulated pathways responsible for the anti-diabetic effects of the supplementation can be identified. In this review, microalgae supplementations have been shown to alleviate diabetes mellitus mainly due to their antioxidant and anti-inflammatory activities that exert protective effects on pancreatic β-cells and subsequent glycemic control. Moreover, the bioactive compounds inhibit enzymes associated with hyperglycemia. A brief summary of the potential therapeutic effects and the mechanisms of microalgae and their bioactive compounds on hyperglycemia has been illustrated in Figure 1. Therefore, the bioactive compound composition and concentration in the supplements are of particular importance to develop them into health supplements. Hence, future studies shall also focus on optimizing the microalgae culture conditions to maximize the yield of bioactive compounds synthesized and standardize the supplements. Furthermore, the methods of inducing T2DM in animal models, such as STZ and/or HFD, can be further refined by considering the dosage of STZ and/or the nutrient composition of HFD to better represent T2DM. Additionally, in order to deem microalgae or their bioactive compounds as safe and efficacious in managing diabetes mellitus, more human studies are needed. Furthermore, upcoming human studies shall include a larger sample size together with a placebo control group to obtain more reliable results and increase the duration of the experiment to reveal the long-term effects of microalgae supplementation.

## Figures and Tables

**Table 1:** Microalgae-derived bioactive compounds and their effects on diabetes mellitus.

Bioactive Compounds	Microalgae Species	Activity on DM
**Vitamins**		
Vitamin E (tocopherols)	*A. platensis* [23,24]	Improves insulin sensitivity [25]
	*N. oculata* [26]	
	*N. gaditana* [27]	
**Fatty acids**		
Linolenic acid	*A. platensis* [23,28] *H. pluvialis* [29,30] *C. vulgaris* [31] *D. salina* [31]	-
Linoleic acid	*A. platensis* [23,28]	Improves insulin secretion [32,33] Improves insulin sensitivity [32,33]
	*N. oculata* [34]	
	*N. gaditana* [35]	
	*D. salina* [36]	
	*H. pluvialis* [30]	
	*P. cruentum* [37,38]	
	*C. vulgaris* [39]	
Arachidonic acid (AA)	*A. platensis* [23,28]	Improves blood sugar level [40,41] Improves glucose tolerance [40] Improves insulin secretion [41] Improves insulin sensitivity [40] Protects pancreatic cells [40]
	*N. gaditana* [35]	
	*H. pluvialis* [30]	
	*P. cruentum* [37,38]	
	*C. vulgaris* [39]	
Eicosapentaenoic acid (EPA)	*A. platensis* [23,28]	Improves blood sugar level [42] Improves insulin sensitivity [42]
	*N. oculata* [34]	
	*N. gaditana* [35]	
	*C. pyrenoidosa* [43]	
	*H. pluvialis* [30]	
	*P. cruentum* [37,38]	
	*C. vulgaris* [39]	
Docosahexaenoic acid (DHA)	*A. platensis* [23,28]	Improves insulin sensitivity [44]
	*C. pyrenoidosa* [43]	
**Pigments**		
Phycocyanin	*A. platensis* [45]	Improves blood sugar level [46] Improves glucose tolerance [47] Improves insulin secretion [47] Improves insulin sensitivity [46] Activates insulin signaling pathway [48] Protects pancreatic cells [47]
	*P. purpureum* [45]	
	*P. cruentum* [49]	
Allophycocyanin	*A. platensis* [45]	-
	*P. purpureum* [45]	
	*P. cruentum* [49]	
Phycoerythrin	*A. platensis* [45]	Improves blood sugar level [50]
	*P. purpureum* [45]	
	*P. cruentum* [49]	
Astaxanthin	*A. platensis* [51,52,53]	Improves blood sugar level [54] Improves glucose uptake [54] Improves glucose tolerance [54] Activates insulin signaling pathway [54] Protects pancreatic cells [55]
	*N. oculata* [34]	
	*N. gaditana* [35]	
	*C. vulgaris* [56]	
	*H. pluvialis* [30,57,58]	
Fucoxanthin	*N. oculata (Total Lipids Content, Lipid Class, and Fatty Acid Composition of Ten Species of Microalgae)* [59]	Improves blood sugar level [60] Activates insulin signaling pathway [60]
Antheraxanthin	*A. platensis* [53]	-
	*N. oculata* [34]	
	*N. gaditana* [35]	
	*C. vulgaris* [56]	
Zeaxanthin	*A. platensis* [53]	Improves blood sugar level [61] Improves glucose tolerance [61]
	*N. oculata* [34]	
	*N. gaditana* [35]	
	*P. purpureum* [62]	
	*P. cruentum* [63]	
	*C. pyrenoidosa* [64]	
	*D. salina* [65]	
Auraxanthin	*N. oculata* [34]	-
	*C. pyrenoidosa* [64]	
Canthaxanthin	*A. platensis* [53]	-
	*N. oculata* [34]	
	*N. gaditana* [35]	
	*C. vulgaris* [65]	
	*H. pluvialis* [65]	
β-cryptoxanthin	*A. platensis* [53]	-
	*P. purpureum*	
	*C. pyrenoidosa*	
	*H. pluvialis*	
Neoxanthin	*N. oculata* [34]	-
	*N. gaditana* [35]	
	*C. pyrenoidosa* [64]	
	*C. vulgaris* [39]	
Violaxanthin	*N. oculata* [34]	-
	*N. gaditana* [35]	
	*C. pyrenoidosa* [64]	
	*C. vulgaris* [39]	
	*H. pluvialis* [58]	
β-carotene	*A. platensis* [28,53]	Improves blood sugar level [66]
	*N. oculata* [34]	
	*N. gaditana* [35]	
	*P. purpureum* [62]	
	*P. cruentum* [67]	
	*C. pyrenoidosa* [64]	
	*C. vulgaris* [64]	
	*D. salina* [68]	
	*H. pluvialis* [58,69]	
Chlorophyll	*A. platensis* [52]	Inhibits α-amylase [70] Inhibits α-glucosidase [70]
	*N. oculata* [34]	
	*N. gaditana* [35]	
	*P. purpureum* [62]	
	*P. cruentum* [67]	
	*C. pyrenoidosa* [43]	
	*C. vulgaris* [56]	
	*H. pluvialis* [58]	
Pheophytins	*N. oculata* [34]	Improves glucose uptake [71]
	*P. purpureum* [62]	
	*C. vulgaris* [39]	
Pheophorbide	*P. purpureum* [62]	Inhibits α-glucosidase [72] Improves blood sugar level [72]
	*C. pyrenoidosa* [73]	
	*C. vulgaris* [39]	
Lycopene	*H. pluvialis* [58]	Improves blood sugar level [74] Improves insulin secretion [74] Protects pancreatic cells [75]
Lutein	*A. platensis* [53]	Improves glucose tolerance [76] Improves insulin sensitivity [77]
	*N. gaditana* [78]	
	*C. vulgaris* [79]	
	*C. pyrenoidosa* [64]	
	*D. salina* [65]	
	*H. pluvialis* [30,58]	
**Carbohydrates**		
Polysaccharides	*A. platensis* [80]	Improves glucose uptake [81] Improves blood sugar level [82,83] Protects pancreatic cells [82]
	*P. cruentum* [84]	
	*C. pyrenoidosa* [85]	
	*C. vulgaris* [65]	
β-glucan	*N. gaditana* [86]	Improves blood sugar level [87] Improves insulin sensitivity [87]
	*P. purpureum* [86]	
**Polyphenols**		
Gallic acid	*A. platensis* [51]	Inhibits α-glucosidase [88] Improves blood sugar level [88] Improves insulin secretion [88] Protects pancreatic cells [88]
Quercetin	*A. platensis* [51]	Inhibits α-glucosidase [89] Improves blood sugar level [89] Improves glucose uptake [90] Improves insulin secretion [91] Improves insulin sensitivity [90] Protects pancreatic cells [91]
	*N. gaditana* [78]	
Apigenin	*A. platensis* [92]	Inhibits α-glucosidase [93] Improves blood sugar level [94] Improves insulin secretion [94] Improves insulin sensitivity [95] Protects pancreatic cells [96]
Catechin	*A. platensis* [51]	Inhibits α-amylase [97] Inhibits α-glucosidase [97] Improves blood sugar level [97]
Caffeic acid	*A. platensis* [51]	Inhibits α-amylase [98] Inhibits β-glucosidase [98] Improves blood sugar level [99,100] Improves glucose uptake [101] Improves insulin sensitivity [100] Improves insulin secretion [101]
	*N. gaditana* [78]	
	*C. vulgaris* [79]	
p-Coumaric acid	*A. platensis* [51]	Improves blood sugar level [102] Improves insulin secretion [103]
	*C. vulgaris* [79]	
Ferulic acid	*A. platensis* [51]	Inhibits α-amylase [104] Inhibits α-glucosidase [104] Improves blood sugar level [105] Improves insulin sensitivity [105] Activates insulin signaling pathway [106] Protects pancreatic cells [107]
	*C. vulgaris* [79]	
Kaempferol	*A. platensis* [51]	Improves blood sugar level [108] Improves insulin secretion [109] Improves insulin sensitivity [108] Protects pancreatic cells [110,111]
Tormentic acid	*A. platensis* [79]	Improves blood sugar level [112] Improves glucose uptake [112] Improves insulin sensitivity [112]
	*N. oculata* [79]	
	*C. vulgaris* [39,79]	
Neophytadiene	*A. platensis* [65]	-
Alphitolic acid	*A. platensis* [79]	-
	*N. oculata* [79]	
	*P. purpureum* [79]	
	*C. vulgaris* [79]	
Maslinic acid	*A. platensis* [79]	Improves blood sugar level [113] Improves insulin sensitivity [113] Activates insulin signaling pathway [114]
	*N. oculata* [79]	
	*P. purpureum* [79]	
	*C. vulgaris* [79]	
Pomolic acid	*A. platensis* [79]	-
	*N. oculata* [79]	
	*P. purpureum* [79]	
	*C. vulgaris* [79]	
Corosolic acid	*A. platensis* [79]	Inhibits α-glucosidase [115] Improves blood sugar level [116] Improves insulin sensitivity [116]
	*N. oculata* [79]	
	*P. purpureum* [79]	
	*C. vulgaris* [79]	
Betulinic acid	*A. platensis* [79]	Inhibits α-amylase [117] Inhibits α-glucosidase [117] Improves glucose uptake [118] Improves insulin secretion [118]
	*N. oculata* [79]	
	*P. purpureum* [79]	
	*C. vulgaris* [79]	
Oleanolic acid	*A. platensis* [79]	Inhibits α-amylase [119] Inhibits α-glucosidase [120] Improves blood sugar level [121] Improves insulin secretion [122] Activates insulin signaling pathway [123] Protects pancreatic cells [123]
	*N. oculata* [79]	
	*P. purpureum* [79]	
	*C. vulgaris* [79]	
Ursolic acid	*A. platensis* [79]	Inhibits α-amylase [124] Inhibits α-glucosidase [124] Improves insulin secretion [125] Protects pancreatic cells [125]
	*N. oculata* [79]	
	*P. purpureum* [79]	
	*C. vulgaris* [79]	
Erythrodiol	*A. platensis* [79]	-
	*N. oculata* [79]	
	*P. purpureum* [79]	
	*C. vulgaris* [79]	
α-Boswellic acid	*A. platensis* [79]	-
	*N. oculata* [79]	
	*P. purpureum* [79]	
	*C. vulgaris* [79]	
Uvaol	*A. platensis* [79]	-
	*N. oculata* [79]	
	*P. purpureum* [79]	
	*C. vulgaris* [79]	
**Bioactive peptides**	*A. platensis* [126]	Inhibits α-amylase [126] Inhibits α-glucosidase [126,127] Inhibits DPP-IV [126]
	*C. vulgaris* [127]	
	*C. pyrenoidosa* [128]	
	*H. pluvialis* [129]	
**Phytol**	*A. platensis* [65] *C. vulgaris* [65]	Improves glucose uptake [130] Activates insulin signaling pathway [130]
**Oxohexadecenoic acids**	*C. karianus* [131,132]	Improves insulin sensitivity [132,133]

**Table 2:** The effects of microalgae and its bioactive compounds in the in vitro studies.

Experiments	Cell Model	Experimental Groups	Finding	Mechanisms	Reference
α-amylase inhibition	-	*Arthrospira platensis* *Nannochloropsis oculata* *Porphyridium purpureum* *Chlorella vulgaris*	All species: Exhibited α-amylase inhibition (Highest in *P. purpureum and N. oculata*)	-	[79]
α- and β-glucosidase inhibition	-	*Synechococcus* sp. GFB01 Conduritol β-epoxide (positive control)	Exhibited high α- and β-glucosidase inhibition	-	[134]
DPP-IV inhibition, α-amylase, and α-glucosidase inhibition	-	Pepsin hydrolysate Ficin hydrolysate Papain hydrolysate Alcalase hydrolysate Aliskiren (positive control)	All hydrolysates: Exhibited DPP-IV inhibition (Highest in alcalase hydrolysates) Limited and α-glucosidase inhibitory capacities	-	[126]
PPARα/γ agonist activity, endogenous PPAR target genes activation analysis, adipocyte differentiation analysis, and adipocyte transcriptomics	COS-1 cells Huh7 cells SGBS pre-adipocyte cells	(7E)-9-OHE or (10E)-9-OHE Rosiglitazone or pirinixic acid (positive controls) Palmitic acid or DMSO (negative controls)	Exhibits PPARα/γ agonist activities Activation of fatty acid catabolism Improvement in adipocyte insulin sensitivity	↑ CPT1A, ACSL3, PLIN1, and ANGPTL4 gene expressions ↑ Adiponectin and leptin gene expressions ↓ IL-6, TNF-α, CXCL1, CXCL5, and IL-1B gene expressions ↑ IRS1 and SLC2A4 gene expressions	[132]

ACSL3: acyl-Coa synthetase long chain family member 3; ANGPTL4: angiopoietin-like 4; CPT1A: carnitine palmitoyltransferase 1A; CXCL1: CXC motif chemokine ligand 1; CXCL5: CXC motif chemokine ligand 5; DPP-IV: dipeptidyl peptidase IV; IL-1B: interleukin-1 beta; IL-6: interleukin-6; IRS1: insulin receptor substrate 1; OHE: oxohexadecenoic acid; PLIN1: perilipin 1; PPAR: peroxisome proliferator-activated receptor; SLC2A4: solute carrier family 2 member 4; TNF-α: tumor necrosis factor-alpha.

**Table 3:** The effects of microalgae-based supplementations on diabetic animal models.

Animal Model	Diabetes Induction	Microalgae and Doses	Experimental Design	Effects on Diabetes Mellitus	Mechanisms	Reference
Male Wistar rats	Drug: 55 mg/kg of BW of STZ	*Nannochloropsis oculata* (NOM) powder (0, 10, 20 mg/kg BW/day) for 21 days	C–H: Non-diabetic + 0 mg/kg BW/day of NOM (normal control) H-10: Non-diabetic + 10 mg/kg BW/day of NOM H-20: Non-diabetic + 20 mg/kg BW/day of NOM C–D: Diabetic + 0 mg/kg BW/day of NOM (diabetic control) D-10: Diabetic + 10 mg/kg BW/day of NOM D-20: Diabetic + 20 mg/kg BW/day of NOM	C–D, D-10, and D-20: ↑ BW ↓ Serum glucose level ↑ Serum insulin level	C–D, D-10, and D-20: ↑ Serum concentrations of GSH-Px, SOD, and FRAP ↓ MDA ↓ Tissue IL-6, NF-κB, IL-1B, and TNF-α	[153]
Male Kunming mice (6 weeks old)	Diet: High-glucose high-fat Drug: 45 mg/kg BW/ day of D-gal for 1 month followed by 50 mg/kg BW of STZ	*Chlorella pyrenoidosa* polysaccharide powder (150 and 300 mg/kg BW/day) for 4 weeks	Normal rats Diabetic rats Diabetic rats + 90 mg/kg BW/day of metformin Diabetic rats + 150 mg/kg BW/day of CPP (CPPL) Diabetic rats + 300 mg/kg BW/day of CPP (CPPH)	CPPL and CPPH: ↑ BW ↑ Insulin level CPPH: ↑ Glucose uptake	CPPL: ↓ FOXO-1 mRNA and protein expressions ↑ GLP-1R mRNA and protein expressions CPPH: ↑ Pancreas weight ↑ Glucose uptake ↑ SOD in liver ↓ MDA in liver CPPL and CPPH: ↑ CAT and GSH-Px in liver Improved pancreatic architecture ↓ IL-6R mRNA and protein expressions Co-modulation of IL-6R/FOXO-1 and GLP-1R/FOXO-1 ↓ Phenylpyruvic acid	[154]
Male Wistar rats (2 months old)	Drug: 45 mg/kg of BW STZ	10% *Nannochloropsis gaditana* powder/day for 2 months, administered orally	C: normal rats CM: normal rats + 10% *N. gaditana*/day D: diabetic rats (diabetic control) DM: diabetic rats + 10% *N. gaditana*/day	DM: ↑ BW ↓ Serum glucose, HbA1c, TG, cholesterol	DM: ↓ IL-6 and TNF-α ↓ MDA and carbonyl proteins in liver mitochondria, and liver and pancreatic tissues ↑ CAT, GSH, and SOD in liver mitochondria and pancreatic tissues ↑ CAT, GSH, SOD, and GST in liver tissue	[2]
Male db/db mice (8 weeks old)	Genetically diabetic	LY: 1 mg/g lyophilized EPA+DHA/day from *Chlorophyceae* and *Eustigamatophyceae* families for 8 weeks Or MD: 2.0% microalgae EPA+DHA-enriched diet for 8 weeks (ad libitum)	RC: Normal/ diabetic strain + rodent chow LY: Normal/ diabetic strain + LY CO: Normal/ diabetic strain + coconut oil MD: Normal/ diabetic strain + MD	Normal/ diabetic strain LY and MD: No significant changes in blood glucose level	Normal LY: ↑ Total antioxidant capacity in plasma Diabetic LY: ↑ Total antioxidant capacity in adipose tissue and plasma Diabetic MD: ↓ Food intake	[155]
Male Sprague–Dawley rats	Drug: 40 mg/kg of BW STZ	*Porphyridium cruentum* powder (600, 1200, and 1800 mg/kg BW/day) for 14 days Or *Porphyridium cruentum* extracellular polysaccharide (150, 300, and 450 mg/kg BW/day) for 14 days	Group 1: Normal rats Group 2: Diabetic rats Group 3: Diabetic rats + 0.6 mg/kg BW/day of glibenclamide (positive control 1) Group 4: Diabetic rats + 1 mg/kg BW/day of acarbose (positive control 2) Group 5: Diabetic rats + 600 mg/kg BW/day of *P. cruentum* powder Group 6: Diabetic rats + 1200 mg/kg BW/day of *P. cruentum* powder Group 7: Diabetic rats + 1800 mg/kg BW/day of *P. cruentum* powder Group 8: Diabetic rats + 150 mg/kg BW/day of extracellular polysaccharide Group 9: 300 mg/kg BW/day of extracellular polysaccharide Group 10: Diabetic rats + 450 mg/kg BW/day of extracellular polysaccharide	Group 9–10: ↓ Blood glucose level	Group 5–10: ↑ Food intake ↑ Pancreatic β-cell number and granulation Group 6, 7, 9, 10: ↑ Pancreatic islets area	[82]
Male rats	Diet: High-fat high-sucrose chow for 8 weeks	*Chlorella pyrenoidosa* or *Arthrospira platensis* ethanol or water extracts (150 mg/kg BW/day) for 8 weeks	NFD: Control group fed normal fat diet HFHS: Control group fed high-fat high-sucrose chow CP55: HFHS + 150 mg/kg BW/day of *C. pyrenoidosa* ethanol extract CPWE: HFHS + 150 mg/kg BW/day of *C. pyrenoidosa* water extract SP55: HFHS + 150 mg/kg BW/day of *A. platensis* ethanol extract SPWE: HFHS + 150 mg/kg BW/day of *A. platensis* water extract	SPWE, CP55, and CPWE: ↓ FBG SP55, SPWE, CP55, and CPWE: Improvement in glucose tolerance	SP55, SPWE, CP55, and CPWE: Improvement in gut microbiota composition	[43]
Male Wistar rats	Drug: 55 mg/kg of BW STZ	*Nannochloropsis oculata* (0, 10, 20 mg/kg BW/day) for 3 weeks	Healthy NOM-0: Healthy rats + 0 mg/kg BW/day of *N. oculata* Healthy NOM-10: Healthy rats + 10 mg/kg BW/day of *N. oculata* Healthy NOM-20: Healthy rats + 20 mg/kg BW/day of *N. oculata* Diabetic NOM-0: Diabetic rats + 0 mg/kg BW/day of *N. oculata* Diabetic NOM-10: Diabetic rats + 10 mg/kg BW/day of *N. oculata* Diabetic NOM-20: Diabetic rats + 20 mg/kg BW/day of *N. oculata*	Diabetic NOM-10 and Diabetic NOM-20: ↑ BW ↓ Serum glucose, TG, cholesterol, and LDL-C ↑ Serum insulin and HDL-C	-	[156]
Male Wistar rats (2.5 months old)	Drug: 55 mg/kg of BW STZ	*Arthrospira plantensis* extract (10, 20, 30 mg/kg BW/day) for 3 weeks	HC: Healthy control rats DC: Diabetic control rats SH10: Healthy rats + 10 mg/kg BW/day of *A. plantensis* SH20: Healthy rats + 20 mg/kg/day BW of *A. plantensis* SH30: Healthy rats + 30 mg/kg/day BW of *A. plantensis* SD10: Diabetic rats + 10 mg/kg BW/day of *A. plantensis* SD20: Diabetic rats + 20 mg/kg BW/day of *A. plantensis* SD30: Diabetic rats + 30 mg/kg BW/day of S. plantensis	SD20 and SD30: ↓ Plasma glucose, TG, cholesterol, LDL-C	SD20 and SD30: ↑ Zinc, iron, selenium, and copper ↓ TNF-α and 1L-6 ↑ SOD, GSH-Px, and CAT ↓ MDA	[157]
Male db/db mice (8 weeks old)	Genetically diabetic	LY: 1 mg/g lyophilized EPA+DHA/day from *Chlorophyceae* and *Eustigamatophyceae* families for 8 weeks Or MD: 2.0% microalgae EPA+DHA-enriched diet for 8 weeks (ad libitum)	BL: Normal/ diabetic strain baseline (control) RC: Normal/ diabetic strain + rodent chow LY: Normal/ diabetic strain + LY SAT: Normal/ diabetic strain + coconut oil MD: Normal/ diabetic strain + MD	Normal/ diabetic strain LY and MD: No significant changes in blood glucose level	Diabetic MD: ↓ % CD3+ ↓ % CD4+ ↓ % CD8+ ↓ IFN-γ, TNF-α ↑ IL-12 ↑ IL-17A ↓ IL-5 ↑ IL-4, IL-10 ↑ IL-6, TGF-β Diabetic LY: ↓ % CD3+ ↓ % CD8+ ↓ IFN-y, TNF-α ↑ IL-12 ↑ IL-17A ↓ IL-5 ↓ IL-4 ↑ IL-6, IL-10, TGF-β	[158]
Male Albino rats	Drug: 45 mg/kg of BW STZ	*Arthrospira platensis* powder (500 mg/kg BW) twice weekly for 2 months	Healthy rats STZ: Diabetic rats SP: Healthy rats + 500 mg/kg BW, twice weekly of *A. platensis* STZ-SP: Diabetic rats + 500 mg/kg BW, twice weekly of *A. platensis*	STZ-SP: ↓ Serum glucose ↓ HbA1C ↑ Serum insulin	STZ-SP: ↓ MDA and TBARS ↑ GSH ↑ GST activity ↑ SOD and catalase activities ↑ SOD, CAT, and GST mRNA expression ↓ PC mRNA expression ↓ Bax, CASP-3, and TNF-α mRNA expressions ↑ Bcl-2 mRNA expression Prevention of MAPK pathways activation ↓ Vacuolation of β-cells of langerhan’s islets	[159]
Male Wistar rats or Swiss mice	Drug: 40 mg/kg of alloxan	*Arthrospira platensis* (Spi, 25, 50, and 100 mg/kg BW/day) for 5 and 10 days	Diabetic control: Diabetic + distilled water Spi 25: Diabetic + 25 mg/kg BW/day of *A. platensis* Spi 50: Diabetic + 50 mg/kg BW/day of *A. platensis* Spi 100: Diabetic + 100 mg/kg BW/day of *A. platensis* Gli: Diabetic + 5 mg/kg BW of glibenclamide	Spi 50 and Spi 100: ↓ Serum TG and cholesterol Spi 25, Spi 50, and Spi 100: ↓ Serum glucose	Spi 50 and Spi 100: ↓ Improved paw tissue architecture ↓ Neutrophil infiltration in paw tissue Spi 100: ↑ Pancreas islet area ↓ Paw TNF-α in paw tissue	[136]
Male Sprague– Dawley rats (12 ± 2 weeks old)	Drug: 50 mg/kg of BW STZ	*Dunaliella salina* (150 mg/kg BW/day) at 72, 64, 48, 40, and 24 h before sacrifice for 3 days	C: Normal rats D: Diabetic rats CD: Normal rats + 150 mg/kg BW/day of *D. salina* DD: Diabetic rats + 150 mg/kg BW/day of *D. salina*	DD: ↑ BW ↓ TG	DD: ↓ TBARS ↑ GSH + GSSG in liver ↓ GSSG/GSH in liver ↓ α-tocopherol liver ↑ α-tocopherol/lipids in plasma	[160]
Female db/db mice	Genetically diabetic	Astaxanthin from marine microalgae (1.0 mg/mouse/day) for 12 weeks	Normal rats Untreated diabetic rats Treated diabetic rats: Diabetic rats + 1.0 mg/mouse/day of astaxanthin	Treated diabetic rats: ↓ Non-FBG ↑ Serum insulin Improvement in glucose tolerance	-	[55]

Bcl-2: B-cell lymphoma 2; BW: body weight; CASP-3: caspase-3; CAT: catalase; CD3: cluster of differentiation 3; CD4: cluster of differentiation 4; CD8: cluster of differentiation 8; FBG: fasting blood glucose; FOXO-1: forkhead Box O1; FRAP: ferric reducing antioxidant power; GLP-1R: glucagon-like peptide-1 receptor; GSH-Px: glutathione peroxidase; GSH: glutathione; GSSG: glutathione disulfide; GST: glutathione-s-transferase; HbA1c: hemoglobin A1c; HDL-C: high-density lipoprotein cholesterol; IFN-γ: interferon-gamma; IL-10: interleukin-10; IL-12: interleukin-12; IL-17A: interleukin-17A; IL-1B: interleukin-1B; IL-4: interleukin-4; IL-5: interleukin-5; IL-6: interleukin-6; LDL-C: low-density lipoprotein cholesterol; MAPK: mitogen-activated protein kinase; MDA: malondialdehyde; NF-κB: nuclear factor kappa B; PC: pyruvate carboxylase; SOD: superoxide dismutase; STZ: streptozotocin; TBARS: thiobarbituric acid reactive substances; TG: triglyceride; TGF-β: transforming growth factor beta; TNF-α: tumor necrosis factor-alpha.

**Table 4:** The effects of microalgae-based supplementation on diabetic patients.

Human Subject	Microalgae and Doses	Experimental Design	Effects on Diabetes Mellitus	Reference
60 male patients with T2DM (40–60 years old)	*Arthrospira platensis* capsule (1 g and 2 g/day) for 2 months	E_1_: Diabetic patients + 1 g/day of *A. platensis* E_2_: Diabetic patients + 2 g/day of *A. platensis* C: Diabetic control	↓ FBG ↓ Postprandial blood glucose ↓ TG, TC, LDL-C, and VLDL-C	[237]
160 male patients with T2DM (45–60 years old)	*Arthrospira platensis* capsule (1 g/day) for 12 weeks	Group-I: Diabetic control + placebo Group-II: Diabetic patients + diet regimen + 1 g/day of *A. platensis* Group-III: Diabetic patients + diet and drug regimen + 1 g/day of *A. platensis* Group-IV: Diabetic patients + diet, drug, and insulin regimen + 1 g/day of *A. platensis*	↓ FBG ↓ HbA1C ↓ TG, TC, LDL-C ↑ HDL-C	[238]
11 male and 11 female patients with T2DM	*Arthrospira platensis* tablet (2 g/day) for 2 months	Control group: Diabetic patients Treatment group: Diabetic patients + 2 g/day of *A. platensis*	↓FBG ↓ Glycated serum protein↓TG, FFA, TC, LDL-C, and VLDL-C	[239]
40 male and female with T2DM (30–60 years old)	*Arthrospira platensis* powder (14 g/day) for 45 days	Positive control group: Diabetic patients + 500 mg of metformin Treatment group: Diabetic patients + 7 g of *A. platensis* powder	↓ FBG ↓ Postprandial blood glucose level	[240]
50 male and female patients with T2DM (30–60 years old)	*Arthrospira platensis* powder (8 g/day) for 3 months	Control group: Diabetic patients Treatment group: Diabetic patients + 8 g/day of *A. platensis*	↓ FBG	[241]
60 male and female patients with T2DM (25–50 years old)	*Arthrospira platensis* powder (2 g/day) for 3 months	Control group: Diabetic patients + metformin + placebo Treatment group: Diabetic patients + metformin + 2 g/day of *A. platensis*	↓ FBG ↓ HbA1C ↓ TG, TC, LDL-C ↑ HDL-C	[242]

FBG: fasting blood glucose; FFA: free fatty acids; HbA1C: hemoglobin A1c; HDL-C: high-density lipoprotein cholesterol; LDL-C: low-density lipoprotein cholesterol; T2DM: type 2 diabetes mellitus; TC: total cholesterol; TG: triglyceride; VLDL-C: very low-density lipoprotein cholesterol.

## Data Availability

Not applicable.
